# The effect of *Rosa canina* L. and a polyherbal formulation syrup in patients with attention-deficit/hyperactivity disorder: a study protocol for a multicenter randomized controlled trial

**DOI:** 10.1186/s13063-022-06297-7

**Published:** 2022-05-23

**Authors:** Haide Golsorkhi, Mostafa Qorbani, Mohammad Kamalinejad, Saeideh Sabbaghzadegan, Mohsen Bahrami, Mohammad Vafaee-Shahi, Hadi Montazerlotfelahi, Elham Abniki, Majid Dadmehr

**Affiliations:** 1 Department of Traditional Medicine, Research Institute for Islamic and Complementary Medicine, Iran University of Medical Sciences, Tehran, Iran; 2 Department of Traditional Medicine, School of Persian Medicine, Iran University of Medical Sciences, Tehran, Iran; 3 Non-communicable Diseases Research Center, Alborz University of Medical Sciences, Karaj, Iran; 4 Chronic Diseases Research Center, Endocrinology and Metabolism Population Sciences Institute, Tehran University of Medical Sciences, Tehran, Iran; 5 Department of Pharmacognosy, School of Pharmacy, Shahid Beheshti University of Medical Sciences, Tehran, Iran; 6 Researcher of Persian Medicine, Private clinic, Tehran, Iran; 7 Department of Pediatrics, School of Medicine, Iran University of Medical Sciences, Tehran, Iran; 8 Department of Pediatrics, School of Medicine, Alborz University of Medical Sciences, Karaj, Iran; 9 Researcher of Clinical Psychology, Private clinic, Tehran, Iran

**Keywords:** Attention-deficit/hyperactivity disorder, ADHD, Complementary and alternative medicines, Herbal medicine

## Abstract

**Background:**

Attention-deficit/hyperactivity disorder (ADHD) is the most common behavioral disorder in childhood and adolescence. A number of these patients do not respond to the current pharmacological treatments and there may also be drug side effects. This study aims to determine the efficacy and safety of two herbal medicine products, including *Rosa canina* L. (RC) and a polyherbal formulation (PHF) syrup, on the clinical manifestations of ADHD in children and adolescents.

**Methods:**

Ninety ADHD patients based on DSM-5 diagnostic criteria will be randomly assigned equally into three groups: (1) RC syrup + methylphenidate (MP), (2) PHF syrup + MP, and (3) placebo + MP according to the inclusion criteria (30 subjects in each group). The syrup dosage is 5cc every 8 h, and MP will have a stabilized dose for 8 weeks during the study. Moreover, Conner^’^s questionnaires will be completed by the teacher and parents before the intervention and then every 4 weeks. Also, the Child Symptom Inventory-fourth edition (CSI-4) and temperament questionnaires will be completed before the intervention and every 4 weeks until 2 months.

**Discussion:**

This trial is the first experiment to determine the effects of RC and PHF syrups on the clinical manifestations of ADHD in children and adolescents. Our findings provide new insight into the effect of these herbal products on the clinical manifestations of ADHD.

**Trial registration:**

Iranian Registry of Clinical Trials IRCT20190923044855N1. Registered on 14 January 2020. The trial was registered at https://www.irct.ir/.

## Background

Attention-deficit/hyperactivity disorder (ADHD) belongs to a subset of neuropsychiatric disorders in children and adolescents, which is described by persistent impairing symptoms of inattention, hyperactivity, and impulsivity [1]. More than half of these patients exhibit these symptoms during adulthood [2–4]. This disorder is frequently combined with learning problems and also an inability with academic/occupational, familial, and social functioning [1–3, 5, 6]. ADHD carries a high risk of comorbidities such as anxiety, affective disorders, substance abuse, and personality disorders. Also, poor education consequences, a great chance for inoccupation, divorcement, or imprisonment are more diagnosed among them compared to the general population [3, 5]. Moreover, mood disorder is reported in 15–75% of children with ADHD and 25% of cases have anxiety [7]. The prevalence rate of ADHD in children is approximately 8–12% all over the world [3]. Treatment strategies for ADHD mainly include pharmacotherapy and psychological and behavioral therapies individually or in combination. Psychostimulant drugs, especially methylphenidate (MP), are highly efficacious and the primary choice of pharmaceutical medications for ADHD treatment [3, 6, 7]. Stimulant medications effectively improve ADHD symptoms in most of these patients; however, about 30% of them do not respond to this class of drug, and there may also be a wide range of drug adverse effects such as loss of appetite, sleep disturbances, and anxiety. Therefore, many parents seek complementary and alternative medicines (CAMs) such as food recommendations, herbal medicine, homeopathy, and exercise for controlling overactivity and inattention of ADHD [7]. Herbal medicine is the most commonly administered CAMs method for ADHD due to its well-tolerance and also parent’s acceptance [8, 9]. There are several mechanisms of action for these medicinal plants associated with the pathogenesis of ADHD, including anxiolytic and antidepressant effects, improvement of cognitive function that help to increase serotonin levels, and central stimulating [7]. Some herbal medicines may be potentially beneficial for ADHD; however, limited information has been reported on their specific effects on this disease in children. So, clinical studies are needed to support their efficacy and safety.

*Rosa canina* L. (RC) belongs to the Rosacea family, which its different parts such as roots, leaves, and fruits have been used to treat several diseases in traditional medicine for centuries [10]. Preclinical studies have shown that RC has neuroprotective effects when used in combination with other herbs [11, 12]. Moreover, it has been demonstrated that RC extract has anxiolytic properties and can improve recognition memory and depressive-like behavior [13, 14]. Furthermore, we hypothesized that a polyherbal formulation (PHF) syrup that contains *Malus domestica* Borkh., *Ocimum basilicum* L., and *Vitis vinifera* L. extract may have beneficial effects on ameliorating memory and cognitive function and anxiety in children with ADHD.

In an experimental animal model, it is shown that chronic administration of *Malus domestica* Borkh. fruit juice has valuable antidepressant activity [15]. *Ocimum basilicum* L. is an edible herb, which has anxiolytic, sedative, and antidepressant-like effects [16, 17]. Also, its neuroprotective properties, especially the improvement of memory and neurological deficit, have been documented previously [18]. Pharmacological studies displayed that *Vitis vinifera* L. has beneficial effects on cognitive function and neuropsychological status; additionally, it indicates anxiolytic-like activity [19, 20].

Therefore, we designed this randomized clinical trial to evaluate the efficacy and safety of RC and PHF syrups on the clinical manifestations of ADHD in children and adolescents.

## Methods

### Study design

**Fig. 1:**
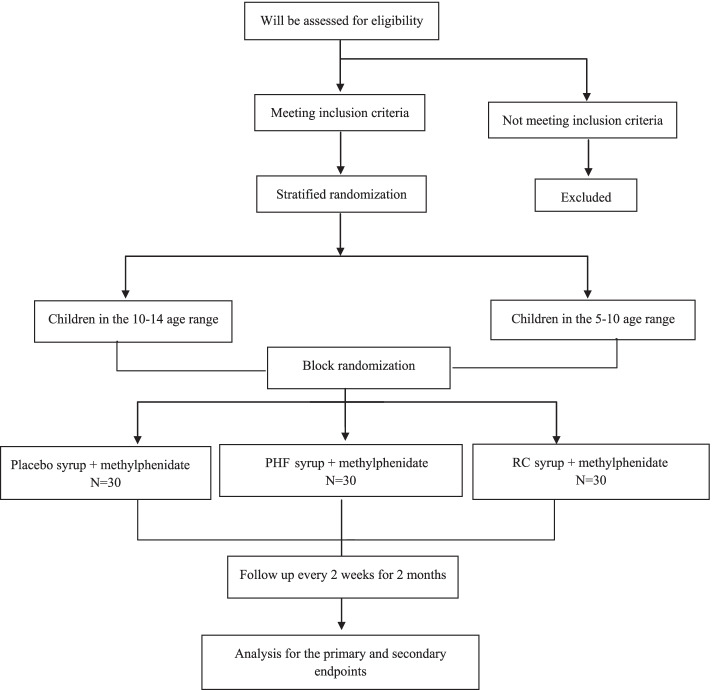
CONSORT flow diagram of the study

**Fig. 2:**
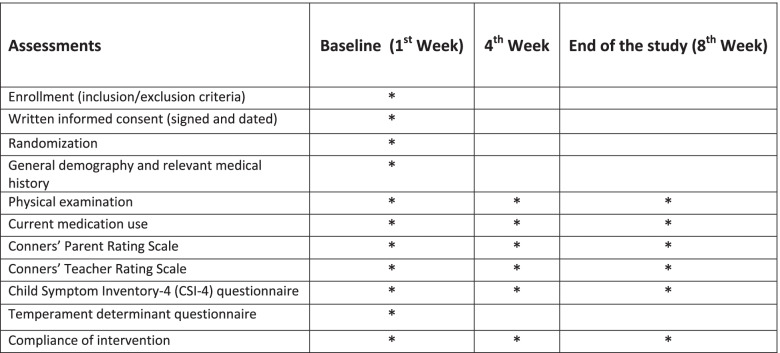
Standard Protocol Items Recommendations for Interventional Trials (SPIRIT) flowchart of the study

This study is a randomized, double-blind, placebo and active product controlled, multicenter clinical trial with three parallel treatment arms to compare the efficacy of two herbal products in children and adolescents with ADHD using the Conners’ Parent and Teacher Rating Scale as a primary outcome measure. Secondary outcome measures are comorbid complaints and side effects. After screening and baseline assessments, 90 patients (aged 5–14 years) will receive one of the three treatments for 8 weeks. Outcome assessments will be performed at the baseline, a 4-week treatment period, and the end of treatment on the 8th week. Figure 1 provides details of the study schedule. The reporting of this trial is conducted according to the Standard Protocol Items: Recommendations for Interventional Trials (SPIRIT) guidelines (Fig. 2). The Consolidated Standards of Reporting Trials (CONSORT) has been used as frameworks of methodology for the design of this protocol.

The study protocol was reviewed and approved by the Medical Ethics Committee of Iran University of Medical Sciences [session no: IRIUMS.REC. 1398.561] and registered in the Iranian Registry of Clinical Trials [registration code: IRCT20190923044855N1].

### Study population

The patients (the children and adolescents aged 5 to 14 years) will be mainly recruited from the three pediatric neurology and psychiatric clinics located at the Rasoul Akram academic hospital, the Firooz Abadi academic hospital, and community clinics in Tehran, Iran. Patients meeting eligibility criteria will be done thoroughly and written consent of the legal representative to participate in the trial will be taken before inclusion. Participants will be randomized, stratified by trial center, to one of the three treatment arms (placebo, RC, and PHF) by randomization software (http://www.randomization.com). The principal investigator (PI) will be situated in each of the study centers to evaluate the implementation of the plan, follow up patients, and deliver checklists and receive and review them and for data collection and entry, as well as respond to patient problems and possible side effects of the drug. Those who meet the inclusion criteria will enroll in the trial and will be randomly allocated into three groups with a 1:1:1 randomization ratio.

### Eligibility criteria

Patients will enroll in this trial if they have these criteria:

(1) Male and female children aged 5 to 14 years; (2) parents and children willing and able to follow all study visits; (3) diagnosis of ADHD, according to the DSM-5 diagnostic criteria; (4) treatment with a stabilized dose of oral stimulant medications (e.g., MP) through the study period; and (5) non-use of other alternative and complementary medications that may interact with the herbal product.

### Exclusion criteria

Children with any of the following criteria will be excluded from the trial:

(1) History of mental retardation; (2) history of bipolar disorder, psychosis, severe conduct disorder, and autism; and (3) history of neurological diseases, seizures, or other serious medical conditions.

### Withdrawal criteria

The study withdrawal criteria are listed as follows:

(1) Parents’ or children’s unwillingness to continue treatment or refusal to participate in the study due to immigration; (2) possible adverse events of treatment; and (3) developing another illness or not taking or misusing the medicine.

The strategies we will use to achieve adequate participant enrolment to reach the target sample size are as follows: educate parents and their children about the trial before asking for consent, make sure they are well aware of the trial, provide written information to parents and children (if required), take the time to answer their questions, and encourage them with regular meetings (face-to-face or by phone) to solve the issues.

The strategies we will use to promote participant retention and complete follow-up are as follows: take the time to answer their questions, encourage them with regular meetings (face-to-face or by phone) to solve the issues, schedule follow-up visits to coincide with routine visits to the office or clinic and facilitate patients’ preferences, and make every effort to locate lost patients.

Participants who withdraw from the trial will be followed up, according to the routine clinical practices.

### Sample size calculation

The sample size was determined according to the previous study [21] using two-mean comparison formula. By considering type I error (0.05) and type II (0.2) and the mean change (SD) of the clinical scale of ADHD score in treatment and placebo groups ( −2.8 ±1.5 and −1.2 ±2.5, respectively) and the attrition rate of 15%, the sample size was calculated 30 subjects in each group (a total of 90 patients). Patients will be allocated to groups using the block randomization method.

### Randomization

The subjects who meet the eligibility criteria will be randomly divided into the intervention and placebo groups using the permuted block randomization method. Moreover, stratified randomization will be used to match the subjects based on the age distributions (5–10 and 10–14 years old). Participants will be randomly assigned to three treatment groups of (1) RC syrup + MP, (2) PHF syrup + MP, and (3) placebo + MP. Then, they will be followed up for 8 weeks. In this trial, random allocation will be performed according to Sealed Envelope tools, by a study statistician through randomization software. Only the person who is located in the selected center will give the patient medicine based on the random allocation sequence list, which will be made.

### Blinding

Both investigators and participants will be blinded to the study design during the implementation of the plan. A sequence allocation list is used to reveal a participant’s allocated intervention during the trial. The unblinding circumstances will be executed in a life-threatening situation like shock or severe drug reaction.

### Intervention

RC, PHF, and placebo are in the form of syrup and will be provided by a botanist in the herbarium center of the pharmacy school, Shahid Beheshti University of Medical Sciences, Tehran, Iran. RC contains *Rosa canina* L. extract; PHF contains *Malus domestica* Borkh., *Ocimum basilicum* L., and *Vitis vinifera* L. extract; and placebo is considered sucrose.

Ninety children and adolescents with ADHD will be invited to the study and randomly assigned to three groups. Subjects will be required to consume 5 cc per day every 8 h according to the recommended dosage for children in the syrup brochure.

The syrups will be prepared in 250 cc glassware and will suffice for about a month. The syrups are identical in size, color, and shape. Any possible complications regarding the numbers of syrups and package will be recorded. Furthermore, the study progress will be pursued by recruiting the subjects every 4 weeks (Fig. 2).

### Adherence

At the first visit, the participants will receive interventions and will be asked to bring all bottles of eaten syrups every month. Returned supplements will be observed to evaluate the level of compliance and adherence to the intervention.

### Patient safety

All participants will be monitored by PI and any probable adverse events (AEs) will be recorded in the case report form (CRF) carefully, during the study period. Furthermore, at the first visit, the phone number of the PI will be given to each participant and it will also be written on the syrup label. The herbal medicines in this study do not have significant side effects, but in case of an adverse event, the PI will collect, report, and manage any adverse events to compensate them from trial participation.

### Study outcomes

#### Primary outcomes

The primary objective of this research is to determine the efficacy and safety of these herbal medicine products on Conner’s score from baseline to 8 weeks after inclusion (including attention and activity function levels and socio-educational functioning levels).

#### Secondary outcomes

The secondary outcomes of this study include the occurrence of adverse events, changes in sleep score, appetite habitus, and comorbidities (e.g., anxiety, depression, and obsessive-compulsive disorders) at the end of the study in comparison with the baseline values.

#### Procedure

At the beginning of the study, goals, methods, and benefits of the trial will be clarified to the parents and participants and an informed consent form will be provided to them. Conners Comprehensive Behavior Rating Scales (Conners CBRS) will be used for obtaining information about several important domains of participant’s behavior, including behavioral, communal, educational topics, and their symptoms. The validity and reliability of this questionnaire were approved previously [22, 23]. In this study, two of Conner’s CBRS questionnaires will be completed (both parent and teacher rating scales):

1. Conners’ Parent Rating Scale (CPRS), a tool for effectively collecting parental reports of child behavior problems
2. Conners’ Teacher Rating Scale (CTRS), a tool for obtaining teacher reports of children’s behavior in the classroom [24–27]

In addition to these questionnaires, the Child Symptom Inventory-4 (CSI-4) questionnaire, which was validated previously [28, 29], will be used to evaluate attention problems in the study participants. The CSI-4 questionnaire is a behavior rating scale that is used for the diagnosis and also severity assessment of ADHD in children between 5 and 12 years old [30, 31]. The temperament determinant questionnaire is also another validated questionnaire used in this study [32].

These questionnaires will be completed before the intervention and then Conner’s questionnaires will just be used every 4 weeks by the teacher and parents during the study. The interviewer will evaluate children signs and symptoms every 4 weeks for 2 months. There is no specific intervention to change the participants’ lifestyle (diet, sleep, activity, etc.).

### Lifestyle changes

No intervention is made in the patient’s living conditions to evaluate the drug’s efficacy.

Patients will not be deprived of their daily treatment (MP/Ritalin). In addition, they will use one of the interventions. To prevent drug interactions between Ritalin and herbal syrups, the patients will consume the syrups 2 h after taking MP/Ritalin. Patients are not allowed to use other herbal medicines during the trial. We will urge patients to drink the syrup three times a day.

### Data management

Data will be managed jointly by the study centers. All the data will be recorded with printed and electronic case report forms (CRFs). After each assessment, the identifiers (e.g., name and birth date) will be anonymized, coded, and stored on a secure server. The files will be backed up on a password-protected computer. The project team designed and prepared the trial and will disseminate the results. The team will meet regularly to discuss the progress of the study. Only outcome assessors have access to CRFs and will perform the double-data entry.

### Data analysis

The normal distribution of continuous variables will be assessed using the Kolmogorov-Smirnov test. Continuous and categorical variables will be presented as mean (standard deviation) and number (%), respectively. The chi-square or Fisher’s exact test will be used to analyze the qualitative variables. Continuous baseline characteristics across the study groups will be tested using the ANOVA test. Missing data will be imputed using the multiple regression imputation method. A two-way repeated measures ANOVA (RMA) will be employed to assess the effect of intervention using time-group interaction. The significance level will be considered less than 0.05. Data will be analyzed using SPSS 21 statistical software.

### Data accessibility

The final trial dataset will be only available to the PI and study statistician (MQ), and other investigators will have limited access. Finally, the study results will be presented only in the publication. Moreover, data can be shared after a reasonable request to the corresponding author.

## Discussion

To the best of our knowledge, this is a novel multicenter study designed for the first time to evaluate the therapeutic effects of two herbal formulations on the treatment of children and adolescents with ADHD and also their comorbidity, appetite, and sleep.

The long-term use and side effects of conventional medications are a concern for parents, so they are increasingly looking for treatment options of CAMs [7]. A number of patients with ADHD diagnosis are frequently using CAMs, including dietary modifications, nutritional supplementation, and just herbal medicine alone or along with current pharmacological treatments in order to manage hyperkinetic and concentration disorders [2]. Diet, exercise, and nutritional supplements all have some potential benefits for a child with ADHD. Herbal remedies, which have been shown to have good effects on restlessness, anxiety, and depression, can also be proper options; however, more research is needed [7]. Moreover, the role of chronic inflammation and oxidative stress has been noted in ADHD. Dietary polyphenols have antioxidant and immunomodulatory properties; so, they may be useful in the management of ADHD [33].

Different herbal preparations have been evaluated in clinical trials as a treatment for children and adolescents with ADHD. The findings propose that some of them may be as effective as MP [6]. RC has some beneficial effects on anxiety, recognition, memory, and depressive-like behaviors [13, 14]. Numerous studies have shown that the ingredients in PHF syrup have considerable ameliorating effects on anxiety, depression, and memory [15–20]. So, the authors hypothesized that RC and PHF syrups will be valuable for the management of ADHD.

## Data Availability

This trial does not involve the storage of biological specimens. The data and materials during the current study can be shared after a reasonable request to the corresponding author. Public access to the full protocol can be accessed as follows: https://www.irct.ir/ (Registration number: IRCT20190923044855N1).

## Abbreviations

ADHD: Attention-deficit/hyperactivity disorder
RC: *Rosa canina* L.
PHF: Polyherbal formulation
DSM-5: Diagnostic and statistical manual of mental disorders
MP: Methylphenidate
CSI-4: Child Symptom Inventory-fourth edition
CAMs: Complementary and alternative medicines
PI: Principal investigator
SD: Standard deviation
CI: Confidence interval
AEs: Adverse events
CRF: Case report form
Conners CBRS: Conners Comprehensive Behavior Rating Scales
CTRS: Conners’ Teacher Rating Scale
CPRS: Conners’ Parent Rating Scale
